# Cancer Cell Migration: Integrated Roles of Matrix Mechanics and Transforming Potential

**DOI:** 10.1371/journal.pone.0020355

**Published:** 2011-05-27

**Authors:** Erin L. Baker, Jaya Srivastava, Dihua Yu, Roger T. Bonnecaze, Muhammad H. Zaman

**Affiliations:** 1 Department of Biomedical Engineering, The University of Texas at Austin, Austin, Texas, United States of America; 2 Department of Chemistry and Biochemistry, The University of Texas at Austin, Austin, Texas, United States of America; 3 Department of Molecular and Cellular Oncology, Cancer Biology Program, The Graduate School of Biomedical Sciences, The University of Texas M.D. Anderson Cancer Center, Houston, Texas, United States of America; 4 Department of Chemical Engineering, The University of Texas at Austin, Austin, Texas, United States of America; 5 Department of Biomedical Engineering, Boston University, Boston, Massachusetts, United States of America; University of Bergen, Norway

## Abstract

Significant progress has been achieved toward elucidating the molecular mechanisms that underlie breast cancer progression; yet, much less is known about the associated cellular biophysical traits. To this end, we use time-lapsed confocal microscopy to investigate the interplay among cell motility, three-dimensional (3D) matrix stiffness, matrix architecture, and transforming potential in a mammary epithelial cell (MEC) cancer progression series. We use a well characterized breast cancer progression model where human-derived MCF10A MECs overexpress either ErbB2, 14-3-3ζ, or both ErbB2 and 14-3-3ζ, with empty vector as a control. Cell motility assays showed that MECs overexpressing ErbB2 alone exhibited notably high migration speeds when cultured atop two-dimensional (2D) matrices, while overexpression of 14-3-3ζ alone most suppressed migration atop 2D matrices (as compared to non-transformed MECs). Our results also suggest that co-overexpression of the 14-3-3ζ and ErbB2 proteins facilitates cell migratory capacity in 3D matrices, as reflected in cell migration speed. Additionally, 3D matrices of sufficient stiffness can significantly hinder the migratory ability of partially transformed cells, but increased 3D matrix stiffness has a lesser effect on the aggressive migratory behavior exhibited by fully transformed cells that co-overexpress both ErbB2 and 14-3-3ζ. Finally, this study shows that for MECs possessing partial or full transforming potential, those overexpressing ErbB2 alone show the greatest sensitivity of cell migration speed to matrix architecture, while those overexpressing 14-3-3ζ alone exhibit the least sensitivity to matrix architecture. Given the current knowledge of breast cancer mechanobiology, these findings overall suggest that cell motility is governed by a complex interplay between matrix mechanics and transforming potential.

## Introduction

The vast majority of breast cancer-related deaths result from metastatic tumors; thus, understanding the interplay between the cellular microenvironment and breast cancer metastatic potential is critically important to the development of effective treatments for this disease. Significant progress has been achieved toward revealing the molecular mechanisms that underlie breast cancer progression [1], [2]; however, quantitative characterization of the associated cellular biophysical attributes remains incomplete. Fundamentally, metastasis proceeds via the migration and invasion of cancer cells through variable extracellular matrix (ECM) environments, and studies have shown that cell migration is indeed sensitive to matrix mechanical properties [3], [4], [5]. Yet, the systems level relationships among matrix mechanics, disease progression, and cell motility in breast cancer are not well understood, especially with respect to physiologically relevant three-dimensional (3D) matrix environments.

Over the past two decades, key breast cancer biomarkers have been identified and linked to specific stages of the disease. Two notable factors are the ErbB2 (HER2/neu) and 14-3-3ζ proteins, both of whose overexpression has been correlated with poor clinical prognoses of breast cancer patients [6], [7]. ErbB2 and 14-3-3ζ have been similarly shown to induce cellular features in vitro that are comparable to clinical presentations. ErbB2 is a transmembrane receptor tyrosine kinase of the epidermal growth factor receptor family of proteins and is involved in multiple signaling pathways that modulate cell growth, differentiation, apoptosis, and other critical cellular processes [8]. Analogously, MECs that are engineered to overexpress ErbB2 have been shown to exhibit hyperplasia and luminal filling in 3D culture, though not full transformation and invasion [9], [10]. ErbB2 is undoubtedly one of the most studied molecules in the field of breast cancer [11] and is a critical target for drug development. In fact, given its ability to confer resistance to certain types of cancer therapy and its prognostic value, determining its status with respect to newly diagnosed breast cancer cases has become a standard practice [12]. Strikingly, the ErbB2 protein is overexpressed in over 50% of early stage non-invasive breast cancers (ductal carcinoma in situ, DCIS) [13]; yet, it is overexpressed in only approximately 25% of later stage invasive and metastatic breast cancers [7]. Explanation of these seemingly inconsistent ErbB2 expression profiles has eluded researchers to date; however, the results reported here suggest that this phenomenon may be explained in part by a mammary epithelial cell (MEC) sensitivity to matrix architecture.

The 14-3-3ζ protein belongs to a larger family of seven 14-3-3 regulatory proteins that are widely expressed and involved in a variety of cellular homeostatic processes, including a general cell survival/anti-apoptotic mechanism [14]. It has been shown that overexpression of 14-3-3ζ confers MECs in 3D culture with a significant resistance to anoikis [15] (a type of apoptosis that occurs when epithelial cells detach from extracellular ligands), which promotes luminal filling and drives MECs towards transformation [16]. Overexpression of 14-3-3ζ has also been shown to induce notable morphological features of epithelial-mesenchymal transition, which are characteristic of progression towards an invasive phenotype [9], [15], [17]. Moreover, analyses of patient biopsies indicate that over 40% of metastatic breast cancers overexpress this protein [6]. Despite their abilities to bestow non-transformed cells with oncogenic attributes, overexpression of neither ErbB2 nor 14-3-3ζ alone is sufficient to confer a complete transformation in vitro. However, their cooperative overexpression has been shown to promote progression from non-invasive carcinoma to invasive cancer in vitro and is also associated with progression of DCIS to invasive and metastatic breast cancer in patients [9].

Given previously established correlations between breast cancer biomarkers and metastatic progression, as well as the current knowledge of substrate-dependent cell motility and cell-matrix interactions, the following fundamental questions remain unanswered for individual MECs with respect to matrix mechanics: (1) Is MEC motility responsive to 3D matrix stiffness? (2) Is this responsiveness related to transforming potential? And (3), is there a relationship between cell motility and transforming potential, given a determined matrix architecture? In this study, we quantitatively investigated these questions by employing time-lapsed confocal microscopy to investigate the effect of matrix stiffness and architecture on migration speed and persistence of individual MECs that are cultured atop 2D matrices and those that are embedded within 3D matrices of differing elastic moduli. We examined human-derived MECs of varied transforming potential with respect to matrices formulated from native Type I collagen, which is the primary structural component of the mammary stroma. Our studies provide novel insights into breast cancer mechanobiology by demonstrating that matrix stiffness and architecture couple with transforming potential to govern the migratory capabilities of MECs.

## Results

**Figure 1:**
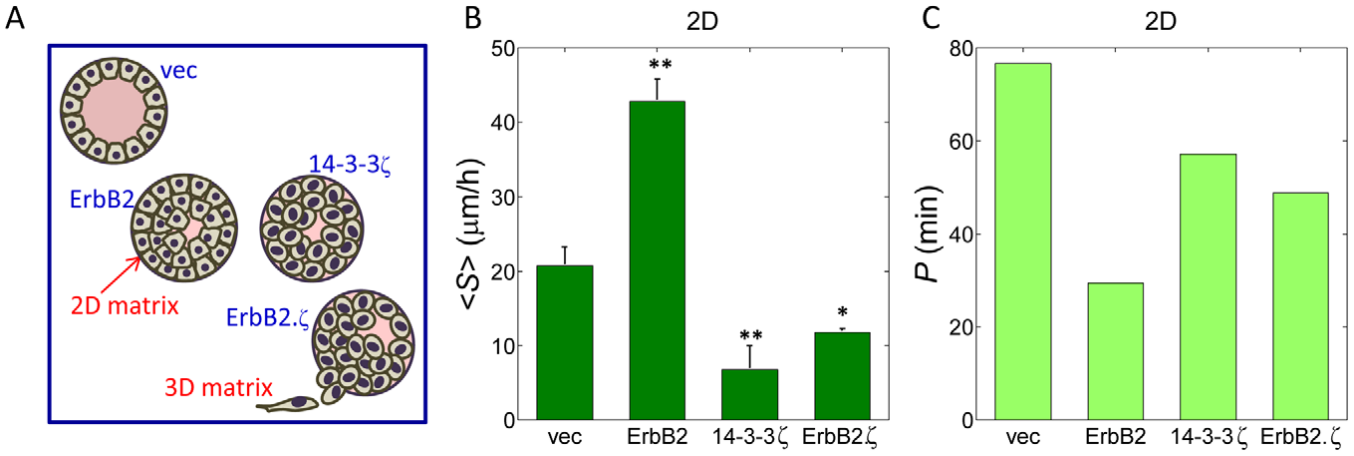
MCF10A cell motility atop 2D matrices. (*A*) Ductal/single lobe cross-sectional depiction of MCF10A breast cancer progression series: 10A.vec (non-transformed), 10A.ErbB2 (partially transformed), 10A.14-3-3ζ (partially transformed), and 10A.ErbB2.ζ (fully transformed). (*B*) Mean cell migration speed <*S*> atop 2D matrices; p-values are with respect to <*S*> of 10A.vec cells. (*C*) Cell population persistence time *P* atop 2D matrices (average *R* = 0.87). All p-values (*,p≤0.05; **, p≤0.01; ***, p≤0.001) determined from *t*-tests for unpaired samples.

In order to explore the relationships between breast cancer transforming potential and cell motility with respect to matrix mechanics, we analyzed a well characterized cancer progression series established from the non-transformed, human-derived MCF10A cell line. We examined four MCF10A sublines, whose extent of transforming potential is characterized according to their growth traits and morphological features when forming acinar structures in 3D culture [9]. As described previously [9], [17], the sublines (Fig. 1*A*) consisted of [1] 10A.vec—a non-transformed control cell line, [2] 10A.ErbB2—a hyperplastic, apoptosis-resistant partially transformed cell line that overexpresses ErbB2, [3] 10A.14-3-3ζ—a depolarized, apoptosis-resistant, and morphologically abnormal partially transformed cell line that overexpresses 14-3-3ζ, and [4] 10A.ErbB2.ζ—an invasive, fully transformed cell line that overepxresses both ErbB2 and 14-3-3ζ.

### Effect of transforming potential on cell motility atop 2D matrices

Cell motility was first examined with respect to the 2D matrix architecture, which is analogous to the MEC layer that lines the inner surface of the ductal basement membrane at the initial stage of invasion into the underlying collagen I-rich stroma in vivo (Fig. 1*A*). In this environment, MECs overexpressing ErbB2 alone migrated with the fastest average migration speed <*S*> (Fig. 1*B*). Non-transformed cells exhibited the second highest degree of motility, followed by sublines overexpressing 14-3-3ζ (Fig. 1*B*). Two-dimensional motility patterns of partially transformed and fully transformed cells are also consistent with transwell motility behavior that has been reported previously: 10A.ErbB2 cells moved with the highest speeds, followed by 10A.ErbB2.ζ and then by 10A.14-3-3ζ [9]. Persistence time *P* (obtained from curve fitting to the persistent random walk model [18], see Materials and Methods) of non-transformed cells was greater than that of all sublines possessing transforming potential (Fig. 1*C*).

### Effect of transforming potential on cell motility within 3D matrices

**Figure 2:**
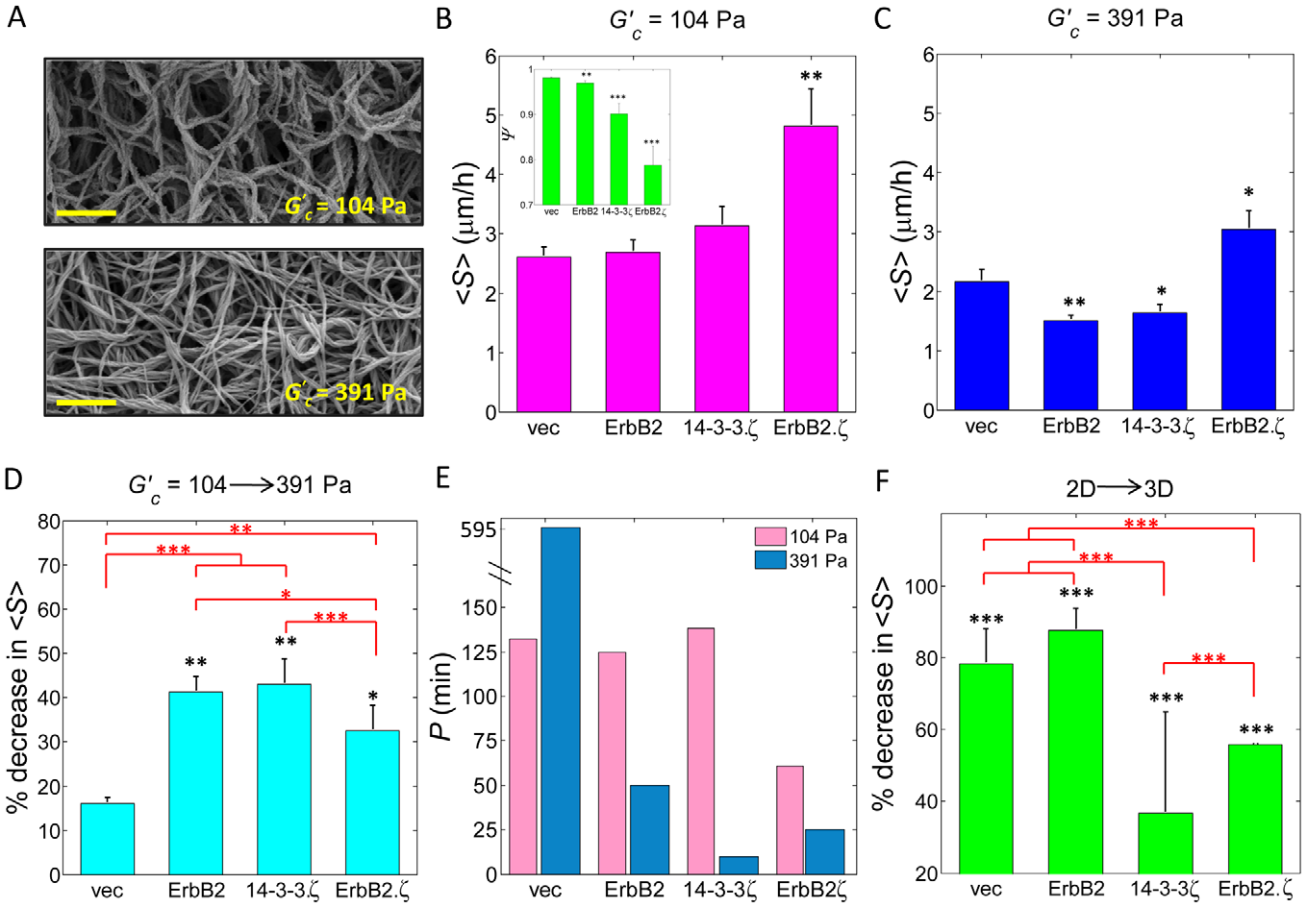
MCF10A cell motility in 3D matrices. (*A*) Scanning electron micrographs of compliant (104 Pa) and stiff (391 Pa) 3D matrices; scale bar is 2 µm. (*B*) Mean cell migration speed <*S*> in compliant 3D matrices. (Inset) cell sphericity *Ψ* as taken from Baker et al. [17]; p-values are with respect to <*S*> (and *Ψ*) of 10A.vec cells. (*C*) Mean cell migration speed <*S*> in stiff 3D matrices; p-values are with respect to <*S*> of 10A.vec cells. (*D*) Percent decrease in <*S*> of cells within compliant matrices relative to cells in stiff matrices. The p-values shown in black reflect the difference in <*S*> between cells within compliant matrices and the same cells within stiff matrices; the p-values shown in red reflect the difference in % decrease in <*S*> among the sublines. (*E*) Cell population persistence time *P* in compliant and stiff 3D matrices (average *R* = 0.87). (*F*) Percent decrease in <*S*> of cells atop 2D matrices relative to cells within compliant 3D matrices. The p-values shown in black reflect the difference in <*S*> between cells atop 2D matrices and the same cells within compliant 3D matrices; the p-values shown in red reflect the difference in % decrease in <*S*> among the sublines. All p-values (*,p≤0.05; **, p≤0.01; ***, p≤0.001) determined from *t*-tests for unpaired samples.

Next, cell motility was assessed with respect to the 3D matrix architecture, which is analogous to the in vivo environment where genetically altered (partially or fully transformed) cells have invaded their local basement membrane and penetrated into the underlying stroma (Fig. 1 *A*). Cell motility assays showed that transforming potential had a notable effect on migration speed <*S*> within relatively compliant 3D matrices (Fig. 2 *A,* stiffness *G^′^_c_* = 104 Pa). Non-transformed MECs (10A.vec) exhibited the slowest average speed, whereas fully transformed MECs (10A.ErbB2.ζ) migrated with the fastest speed (Fig. 2*B*). MECs overexpressing ErbB2 or 14-3-3ζ alone, although partially transformed, did not show a notable change in motility relative to 10A.vec cells (Fig. 2*B*) in compliant 3D matrices. Furthermore, migration speed <*S*> in compliant matrices negatively correlated with the sphericity cell morphology index *Ψ* that we previously reported of these sublines when cultured in the same 3D matrices [17]. As shown in Fig. 2*B* (inset) [17], *Ψ* decreases as <*S*> increases, according to MEC transformation profile. In compliant matrices, cell population persistence time *P* was lowest for fully invasive cells (Fig. 2*E*).

### Effect of 3D matrix stiffness on cell motility

In relatively stiffer 3D matrices (Fig. 2*A*, stiffness *G^′^_c_* = 391 Pa), cell motility assays revealed a behavior significantly different from that observed in compliant matrices. In the stiffer matrix environment, fully transformed MECs migrated faster than all other sublines (Fig. 2*C*). However, partially transformed cells (10A.ErbB2 and 10A.14-3-3ζ) migrated notably slower than both non-transformed and fully transformed cells. The shift in cell motility between compliant and stiff matrices is further displayed as a percent decrease in migration speed, according to transformation profile (Fig. 2*D*); this depiction shows that among the sublines whose migration speed was sensitive to 3D matrix stiffness, the motility of fully transformed cells was least affected by the increase in matrix stiffness. As compared to cells in compliant 3D matrices, cell persistence time *P* in stiff 3D matrices (Fig. 2*E*) was lower for cells possessing partial or full transforming potential, but notably higher for non-transformed cells (10A.vec).

### Integrated effects of matrix architecture, matrix stiffness, and transforming potential on cell motility

Comparing migration speeds of cells atop 2D matrices to those embedded within similar 3D matrices shows that matrix architecture has a significant effect on cell motility. Figure 2 *F* depicts this shift in motility as a percent decrease in speed of cells atop 2D matrices as compared to those within compliant (104 Pa) 3D matrices. Indeed, motility in 3D matrices is significantly reduced for all cell sublines examined; however, of the sublines possessing partial or full transforming potential, cells overexpressing ErbB2 alone (10A.ErbB2) showed the greatest sensitivity to matrix architecture. The 10A.ErbB2 subline experienced a significant 94% decrease (15-fold reduction) in cell migration speed when in 3D matrices as compared to that when these cells were attached to 2D matrices.

**Figure 3:**
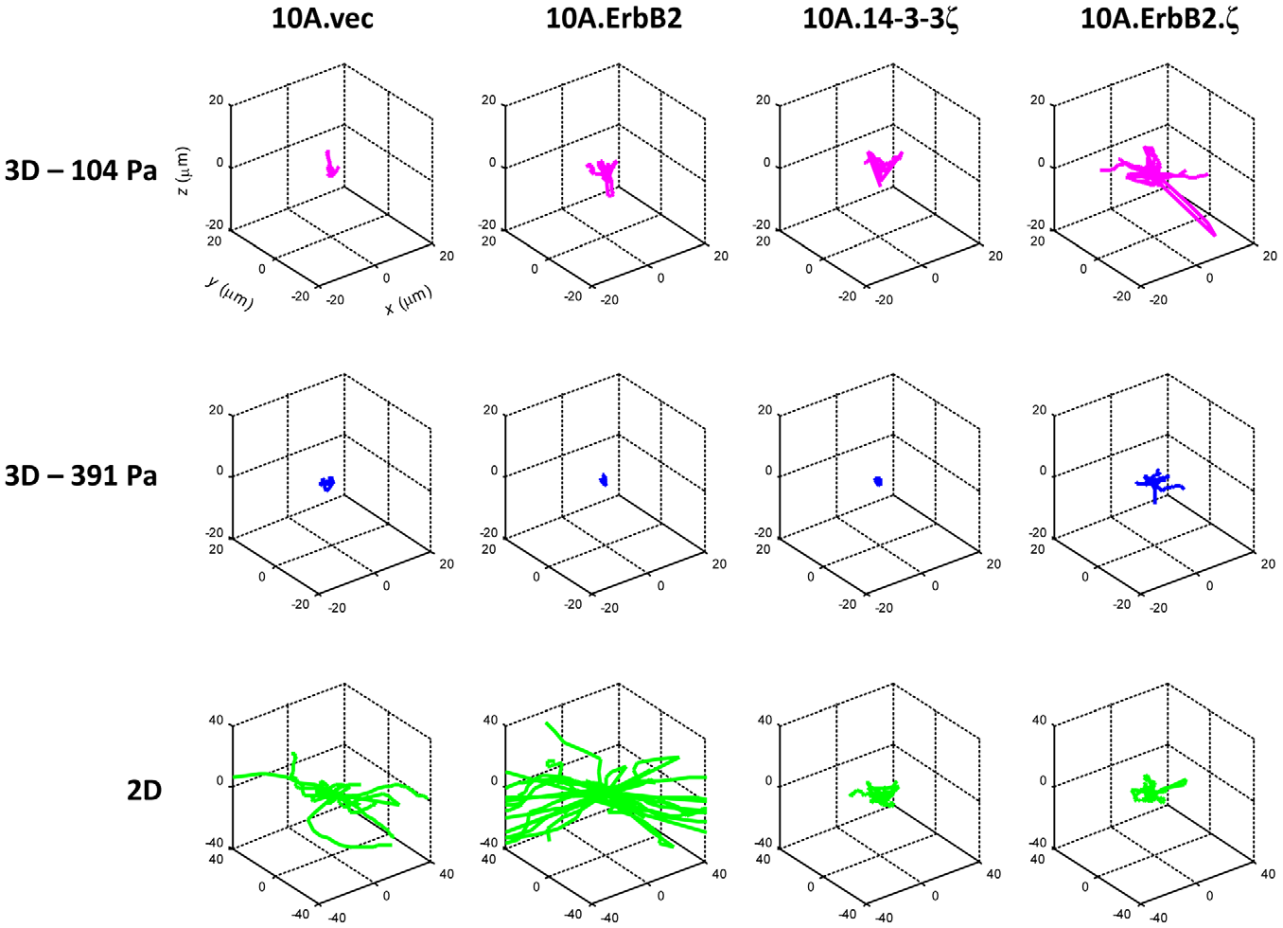
Windrose plots of MCF10A cell migration. Top row lists cell line; left column lists matrix condition. Cells in 2D matrices exhibited the highest degree of motility, followed by cells within compliant (104 Pa) 3D matrices and then by cells within stiff (391 Pa) 3D matrices.

**Figure 4:**
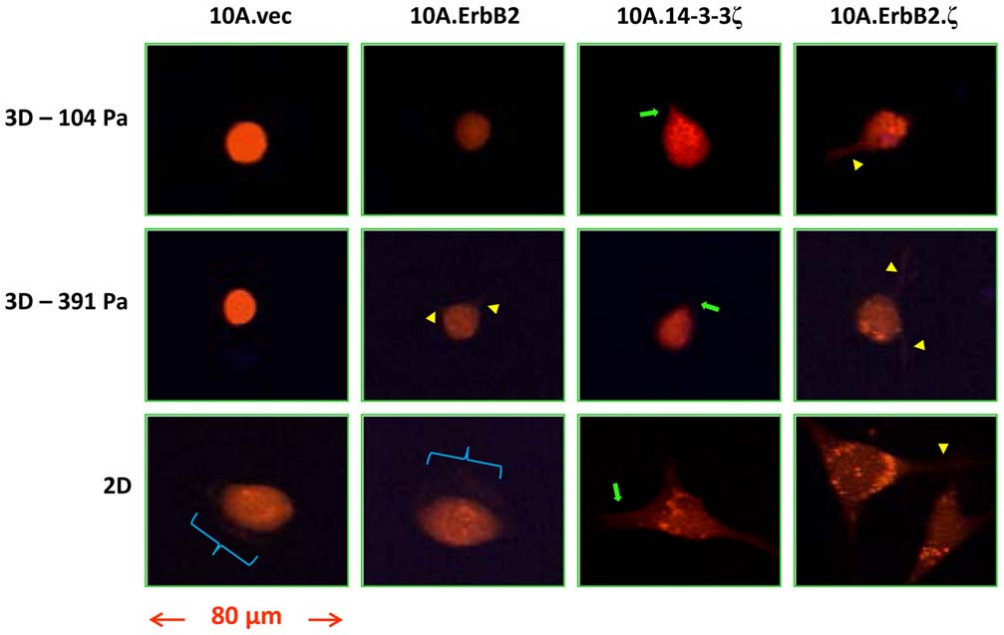
Single-plane confocal images of MCF10A cells embedded within 3D Type I collagen matrices and cultured atop 2D Type I collagen matrices. Top row lists cell line; left column lists matrix condition. Yellow carats indicate thin, rod-like cellular processes. Green arrows indicate tubular-shaped cellular protrusions. Blue brackets indicate sheet-like cellular protrusions.

Examination of 3D Windrose plots (Fig. 3) provides a broad, summary view of the migratory character exhibited by this MCF10A progression series (rows represent matrix condition, while columns represent subline). *XY*-plane confocal images (Fig. 4) also show typical representative cells and morphological features exhibited by each of the four sublines, which may bear some association to migratory data presented here, as well as cell stiffness findings that we have reported previously [17]. MECs overexpressing 14-3-3ζ alone exhibited tubular-shaped protrusions (Fig. 4, green arrows) [19] across all matrix conditions, while those co-overexpressing both ErbB2 and 14-3-3ζ exhibited thin, rod-like extensions (Fig. 4, yellow carats) for all matrix conditions. Cells overexpressing ErbB2 alone showed minimal rod-like extensions and only when embedded within relatively stiff (391 Pa) matrices, while the remaining MEC sublines displayed similar degrees of protrusion in both compliant and stiff matrices. The fastest migrating cells on 2D matrices (10A.vec and 10A.ErbB2) exhibited sheet-like cellular processes in this environment (Fig. 4, blue brackets).

## Discussion

Cell motility can be influenced by a number of parameters, including extracellular chemical gradients [20], matrix mechanical properties [4], matrix degradation [21], intracellular contractility [5], and cell adhesivity [22]. Increasingly, cancer cells have become the focus of studies that explore the effect of the extracellular environment on cellular homeostasis [4], [23], cellular viscoelasticity [24], [25], and cell motility. While significant progress has been achieved in uncovering some of the molecular mechanisms and signaling pathways that underlie breast and other cancers [8], [14], much less is known about the associated cellular biophysical attributes. It has long been established that breast cancer metastasis is fundamentally executed by cell migration from a primary tumor mass through the underlying stroma and that breast collagen density is correlated with breast cancer progression [26]. Moreover, cancer cell migratory capability can be influenced by the stiffness of the ECM [5]. However, the relationship between the external cellular mechanical environment and the motility of breast cancer cells is not understood. The interplay between these parameters is further confounded by the stage of breast cancer progression and may also bear relation to intracellular mechanical properties [17]. In order to investigate the interplay among matrix mechanics, cell motility, and transforming potential in breast cancer, we have utilized time-lapsed confocal microscopy to gauge the migration speed and persistence of MECs that are attached to 2D matrices and those that are embedded within 3D matrices, both comprised of native Type I Collagen. By examining a breast cancer progression series of sublines that derive from a single MEC parent line, we are able to directly compare kinesis of cells that possess varying transforming potential.

The in vivo extracellular microenvironment is a heterogeneous medium that consists of several components, with the relative balance and significance of these components depending upon the extent of cancer progression. In this study, we have probed the motility of MECs that have the capacity to freely navigate their ECM. For the case of 3D matrices, this is physiologically most comparable to individual MECs that have invaded their local ductal basement membrane and may migrate within the underlying stroma (Fig. 1*A*); for the case of 2D matrices, this is most analogous to early stage cancer cells that may exhibit enhanced motility along the inner ductal basement membrane at the initial stage of invasion into the underlying collagen I-rich stoma (Fig. 1*A*). We examined single cells that are wholly engaged with the matrix (but unattached to other cells) in order to experimentally control the degree and type of cell surface attachment; thus, the MECs examined here form cell-matrix attachments via β1 integrins [27].

Examining cell migration with respect to both 2D and 3D matrices offers a broad perspective of MEC motility (Fig. 3). Overexpression of ErbB2 has been shown previously to bestow MECs with increased proliferative capacity [10], and it has also been associated clinically with early stage breast cancer (DCIS) [9]. In fact, the matrix environment of early stage breast cancers (DCIS) more closely resembles that of a 2D matrix architecture than it does a 3D matrix environment (Fig. 1*A*). When cultured atop 2D matrices, MECs overexpressing ErbB2 alone migrated with the fastest speed (Fig. 1*B*); non-transformed cells exhibited the second highest degree of motility, followed by sublines overexpressing 14-3-3ζ (Fig. 1*B*). The significantly reduced migration speed of 14-3-3ζ-overexpressing sublines relative to the remaining two sublines suggests that 14-3-3ζ-mediated downregulation of E-cadherin [9], [15] may yield a lesser effect on cell motility atop 2D matrices than on MECs that are tasked with navigating a 3D matrix environment. This again underscores the complex interplay between transforming potential and matrix mechanics in governing cell motility. High persistence of non-transformed cells relative to the remaining genetically altered sublines (Fig. 1*C*) indicates that transforming potential may grant MECs with a heightened sensitivity to 2D matrix topography, which would be reflected in randomly changing cell trajectories as compared to more directed cell movements of non-transformed cells. An increased sensitivity to matrix topographical cues should be advantageous to cells that seek to invade their local basement membrane.

Our results suggest that within relatively compliant 3D matrices (104 Pa), transforming potential in association with morphological features are the dominate factors that influence cell motility. As shown in Fig. 2 *B*, fully transformed cells (10A.ErbB2.ζ) migrated with the fastest speed <*S*> in this environment, followed by morphologically altered 14-3-3ζ-overexpressing cells, and then

Comparing MEC motility in compliant matrices to that of MECs in stiffer matrices suggests that cell migratory ability is not simply an effect of transforming potential; rather, it is governed by a balance of intrinsic cell biophysical attributes along with matrix mechanics. The slight enhancement of thin, rod-like extensions exhibited by 10A.ErbB2 cells in stiffer matrices (Fig. 4), although subtle, may bear association to the high stiffness sensing capability that we have previously reported of this subline [17]. However, although morphological features of a given subline were similar in 3D matrices of differing stiffness (Fig. 4), the system-wide migration speed profiles show distinct patterns relative to matrix stiffness (Fig. 2*B* and *C* and Fig. 3). In both matrix environments, non-transformed MECs migrated more slowly than fully transformed MECs, which migrated with the fastest speeds. However, in the stiffer matrices (391 Pa), partially transformed cells (10A.ErbB2 and 10A.14-3-3ζ) migrated significantly slower than non-transformed cells. Increased matrix stiffness resulted in a decreased migratory ability for fully transformed cells, but this effect was even more pronounced for partially transformed cells (Fig. 2*D*). These results suggest that sufficient density-dependant 3D matrix stiffness may play a role in significantly hindering the migratory ability of partially transformed cells; however, this increase in 3D matrix stiffness may not be ample to overcome the aggressive behavior exhibited by fully invasive cells, as evidenced by the only moderate reduction in migration speed of 10A.ErbB2.ζ cells (Fig. 2*D*). Results from our prior study of these sublines showed that in the stiffer matrices, 10A.ErbB2 cells exhibit the highest intracellular stiffness, while fully transformed 10A.ErbB2.ζ cells exhibit a moderate stiffness, and 10A.14-3-3ζ cells exhibit a relatively low stiffness [17]. In total, considering the current motility data in conjunction with the results of our previous investigations suggests that MEC migration in 3D environments proceeds at an optimal balance among genetic transformation profile, intracellular stiffness, advantageous morphological features, and matrix stiffness. Thus, an increase in cell migratory speed that may otherwise result from partial or full transformation may be mitigated in part by density-dependant matrix stiffness.

The final analysis of this study (Fig. 2*F*) presents a very provocative result, given that the ErbB2 oncogene is detected with lower frequency in invasive and metastatic breast cancers than it is in early stage breast cancers. In fact, the prevalence of ErbB2 overexpression in invasive and metastatic breast cancer is only *half* that of early stage cancers [9], which has been a perplexing phenomenon. The results from our motility assays suggest that a shift in matrix architecture may be associated with this behavior. When MECs transition from a non-invasive early stage cancer to an invasive and then metastatic cancer, they migrate from atop a 2D basement membrane surface to within a surrounding 3D stroma and thus experience a shift in matrix architecture (Fig. 1*A*). In this study, the motility of 10A.ErbB2 cells shows the greatest sensitivity to a shift in matrix architecture, as compared to the other sublines that possess partial or full transforming potential (Fig. 2*F*). It follows that cells exhibiting a significantly diminished stromal migratory ability may be less likely to completely invade their local boundaries and further traverse the surrounding stroma to later manifest as metastatic breast cancer. It should also be noted that overexpression of 14-3-3ζ significantly suppressed 2D migration (Fig. 1*B*), while synergistically enhancing 3D migration when ErbB2 was also overexpressed (Fig. 2*B* and *C*). Thus, the motility of cells overexpressing 14-3-3ζ alone exhibited the least sensitivity to matrix architecture, as compared to 10A.ErbB2 and 10A. ErbB2.ζ cells (Fig. 2*F*).

In summary, the present study provides novel insights into breast cancer motility by demonstrating that transforming potential couples with matrix stiffness and architecture to influence the migration speed and persistence of MECs. Numerous prior investigations have significantly contributed to the present understanding by examining ErbB2 and 14-3-3ζ-mediated effects on intracellular stiffness [17], MEC motility in soluble chemical gradients [20], cancer cell migration with respect to ligand availability [5], and motility-induced matrix remodeling [28]. By employing time-lapsed imaging, we have added to this knowledge by directly measuring the migration speed of MECs both cultured atop 2D matrices and embedded within 3D matrices. The relationships between breast cancer cell motility and substrate characteristics are complex; further clarification of these connections may arise from additional future studies that examine the effects of 2D matrix stiffness and matrix protein constitution on the motility of the sublines examined here. A clearer understanding of MEC-matrix interactions holds broad promise that may ultimately direct the development of targeted therapies and cancer-focused translational research.

## Materials and Methods

### Cell culture

Motility assays were performed on stable sublines that were established as described previously [9] from the non-cancerous, human-derived MCF10A MEC line (provided by Dr. Robert Pauley of the Karmonos Cancer Institute, Detroit, MI). Cell lines were maintained in 2D monolayer culture in DMEM/F12 growth media [29] within a humidified incubator at 37°C, 5% CO_2_ until the time of experimentation.

### Collagen matrix preparation and characterization

Two-dimensional matrices were created by diluting high concentration Type 1 collagen to 2 mg/mL using 20 µL of ethanol-dialized 2.0 µm carboxylated, yellow-green fluorescent polystyrene tracer beads (Molecular Probes, Carlsbad, CA) (approximately 2% solid) and a balance of 0.01 M HCl; 1.5 mL of the solution was then deposited into the well of a 35 mm glass bottom dish and allowed to incubate at room temperature for 1 h. Following this period, the solution was aspirated, leaving only the bead-impregnated collagen coat that had adhered to the glass bottom (see Fig. S1 *A*). Dishes were then rinsed twice with PBS and stored at 37°C, 5% CO_2_ for 45 min until fluorescently labeled cells were deposited into the dish.

Three-dimensional matrices were formulated from high concentration Type I collagen (BD Biosciences, San Jose, CA), which was diluted to two concentrations of 2 and 4 mg/mL. Equal parts collagen and neutralizing solution (100 mM Hepes in 2X PBS at pH 7.3) were mixed with 20 µL of the bead suspension and a balance of 5×10^5^ fluorescently labeled cells suspended in growth media to achieve the final concentration [30] (see Fig. S1 *B*). Each matrix solution (1 mL) was then deposited across the surface of a 35 mm glass bottom dish (MatTek, Ashland, MA). Matrix solutions were allowed to gel for 90 min at 37°C, 5% CO_2_, upon which 1.5 mL of growth media were deposited atop the 3D matrices to provide cells with adequate nutrients during a subsequent 4.5 h incubation period at 37°C, 5% CO_2_. Matrix stiffness was measured using cone and plate rheometry and quantified in terms of the bulk shear elastic modulus of the collagen gel *G^′^_c_*, which is reported as 104 and 391 Pa for 3D Type I collagen gels of concentration 2 and 4 mg/mL, respectively, as described previously [17]. In this manuscript, matrices of modulus 104 Pa are referred to as relatively compliant, while those of modulus 391 Pa are described as relatively stiff. These elastic moduli are consistent with those reported previously for non-cancerous and breast cancer-associated stroma [31]. Three-dimensional matrices were visualized using a Zeiss Supra 40 VP scanning electron microscope (see Text S1).

### Cell tracking

At the time of experimentation, adherent monolayer cell cultures were stained with fluorescent Cell Tracker Orange CMTMR (Molecular Probes) and subsequently detached using 0.05% Trypsin/0.53 mM EDTA (Cellgro, Manassas, VA). For the 3D matrix assay, cells were imaged following a total incubation period of 6 h within the matrices (see *Collagen matrix preparation and characterization*). Time-lapsed image z-stacks of total thickness 120–150 µm were collected every 10 min for a total of 4 h at a magnification of 20X using the LSM 5 Live (Carl Zeiss, Thornwood, NY) (see Fig. S1 *C*). Z-stack images were collected at intervals of 1.65 µm, as optimally computed by the LSM software. For the 2D matrix assay, 2×10^5^ fluorescently labeled cells suspended in 1.5 mL of growth media were deposited atop the coated glass bottom dish and incubated for 6 h prior to imaging. Time-lapsed image z-stacks of total thickness 25–30 µm were collected as described for the 3D matrix assay. During imaging, both 2D and 3D cultures were housed within a microscope-mounted incubation chamber that was maintained at 37°C, 5% CO_2_.

### Motility analyses

Following image collection, cell trajectories and extracellular bead trajectories were generated using the spots detection and position tracking features of Imaris image analysis software (Bitplane, St. Paul, MN). Extracellular tracer beads were used to track overall sample drift during imaging.Cell migration speed *S* was calculated as the total cell track length divided by the total time over which each cell was recorded in the image field of view. At each time interval, the incremental cell trajectory was corrected by adjusting for the sample drift (computed as the average displacement vector of all tracer beads) that occurred during the same time interval. Thus, sample drift was accounted for and is not reflected in the reported values of average population cell speed <*S*> and population persistence time *P*. Population persistence time was determined by fitting the (adjusted) mean squared cell displacements <*d^2^*(*t*)> to the persistent random walk model [18], 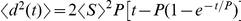, where *t* is the elapsed time. The average correlation coefficient for the random walk curve fits was *R* = 0.87. Three-dimensional Windrose plots display (adjusted) cell tracks for each matrix condition (Fig. 3). Cell motility assays were performed 3–4 times per 3D matrix per condition per cell line and twice per cell line for the 2D matrix architecture. An average total of *N* = 48 cells were imaged per combination of 3D matrix and cell line, with an average of *M* = 125 tracer beads imaged per experiment; an average total of *N* = 61 cells were imaged per cell line for the 2D matrix architecture, with an average of *M* = 6 tracer beads imaged per experiment. All calculations were performed using MATLAB.

## Supporting Information

Figure S1 **Experimental systems utilized for cell motility assays.** (*A*) Illustration of 2D assay; cells were attached to a Type I collagen coat embedded with tracer beads. (*B*) Illustration of 3D assay; both cells and tracer beads were wholly suspended within 3D Type I collagen matrices. (*C*) Maximum intensity projection of confocal z-stack; mammary epithelial cells (orange) and 2 µm tracer beads (green) embedded within a 3D Type I collagen matrix. Tracer beads serve as reference markers to account for global sample drift.
(TIF)

Text S1 (DOC)
