# Supplementary figures and images for: Cancer Cell Migration: Integrated Roles of Matrix Mechanics and Transforming Potential
Source: PLoS One. 2011 May 27;6(5):e20355. doi: 10.1371/journal.pone.0020355 (PMC3103552; doi:10.1371/journal.pone.0020355)

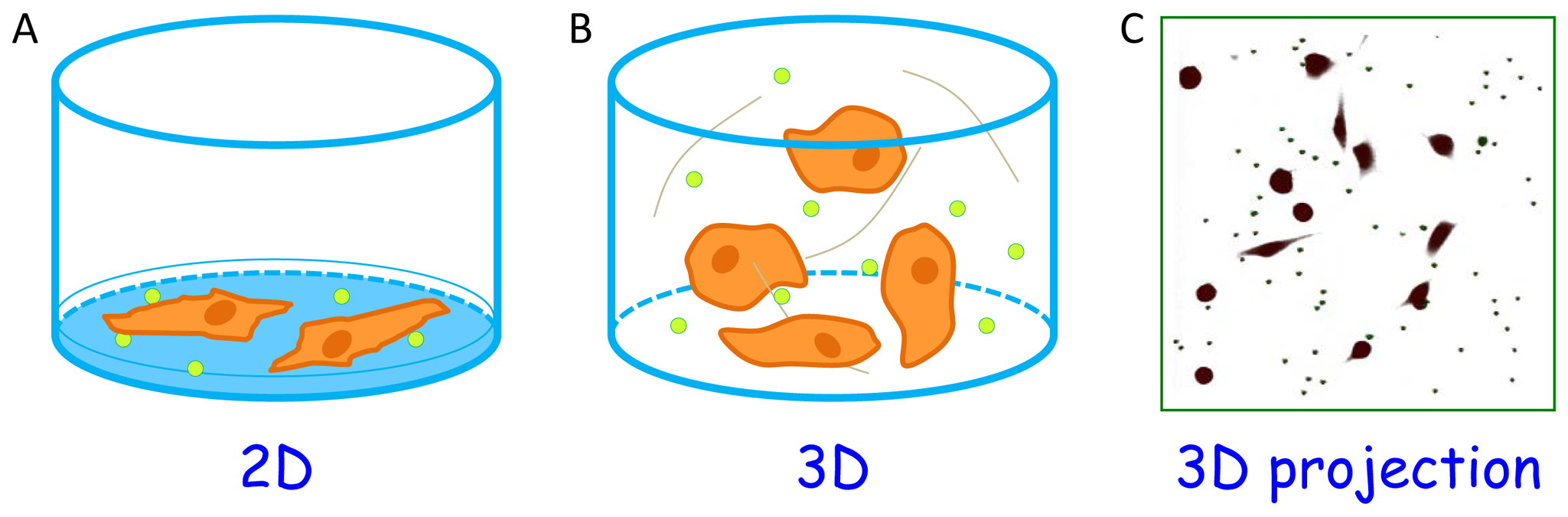

Supplement: Figure S1 — Experimental systems utilized for cell motility assays. (A) Illustration of 2D assay; cells were attached to a Type I collagen coat embedded with tracer beads. (B) Illustration of 3D assay; both cells and tracer beads were wholly suspended within 3D Type I collagen matrices. (C) Maximum intensity projection of confocal z-stack; mammary epithelial cells (orange) and 2 µm tracer beads (green) embedded within a 3D Type I collagen matrix. Tracer beads serve as reference markers to account for global sample drift. (TIF) [file pone.0020355.s001.tif]
