# Supplementary material for: Cancer Cell Migration: Integrated Roles of Matrix Mechanics and Transforming Potential
Source: PLoS One. 2011 May 27;6(5):e20355. doi: 10.1371/journal.pone.0020355 (PMC3103552; doi:10.1371/journal.pone.0020355)
Supplement: Text S1 — (DOC) [file pone.0020355.s002.doc]

**Supplemental Materials and Methods**

*Scanning electron microscopy visualization of 3D matrices*

Three-dimensional matrices (Fig. 2 *A*) were visualized using scanning electron microscopy (SEM). Briefly, following matrix polymerization, gels were fixed and stained using tannic acid, gluteraldehyde, and osmium tetroxide (TAGO) fixation. One percent tannic acid and 0.8% gluteraldehyde were suspended in 0.2 M cacodylate buffer; 1 mL of this solution was used to fix each 1 mL gel sample for 2 h at room temperature. Gels were rinsed twice with 500 mL of 0.2 M cacodylate buffer for 15 min each. Each sample was then post-fixed with 1 mL of 2% OsO_4_ prepared in 0.2 M cacodylate buffer for 1 h at 4°C. Samples were then dehydrated using ethanol exchange; each exchange occurred in 15 min increments at room temperature using the following gradient: 1 x 50%, 1 x 75%, 1 x 90%, and 2 x 100% absolute ethanol. Samples were then subjected to HMDS exchange; each exchange was performed for 10 mins using the following gradient scheme: 2 x 50% ethanol/50% HMDS, 2 x 100% HMDS. Samples were finally sputter coated with a 15 nm layer of Pt/Pd and imaged using a Zeiss Supra 40 VP scanning electron microscope.
